# Post-Traumatic Headache: A Review of Prevalence, Clinical Features, Risk Factors, and Treatment Strategies

**DOI:** 10.3390/jcm12134233

**Published:** 2023-06-23

**Authors:** Ioannis Mavroudis, Alin Ciobica, Alina Costina Luca, Ioana-Miruna Balmus

**Affiliations:** 1 Department of Neuroscience, Leeds Teaching Hospitals, NHS Trust, Leeds LS2 9JT, UK; i.mavroudis@nhs.net; 2 Faculty of Medicine, Leeds University, Leeds LS2 9JT, UK; 3 Department of Biology, Faculty of Biology, Alexandru Ioan Cuza University, 700506 Iasi, Romania; 4 Centre of Biomedical Research, Romanian Academy, B dul Carol I, No. 8, 700506 Iasi, Romania; 5 Academy of Romanian Scientists, Splaiul Independentei nr. 54, Sector 5, 050094 Bucuresti, Romania; 6 Department of Mother and Child, Medicine-Pediatrics, “Grigore T. Popa” University of Medicine and Pharmacy, 16, Universitatii Street, 700115 Iasi, Romania; 7 Department of Exact Sciences and Natural Sciences, Institute of Interdisciplinary Research, “Alexandru Ioan Cuza” University of Iasi, 700057 Iasi, Romania

**Keywords:** post-traumatic headache, mild traumatic brain injury, post-concussion syndrome

## Abstract

Post-traumatic headache (PTH) is a common and debilitating consequence of mild traumatic brain injury (mTBI) that can occur over one year after the head impact event. Thus, better understanding of the underlying pathophysiology and risk factors could facilitate early identification and management of PTH. There are several factors that could influence the reporting of PTH prevalence, including the definition of concussion and PTH. The main risk factors for PTHs include a history of migraines or headaches, female gender, younger age, greater severity of the head injury, and co-occurring psychological symptoms, such as anxiety and depression. PTH clinical profiles vary based on onset, duration, and severity: tension-type headache, migraine headaches, cervicogenic headache, occipital neuralgia, and new daily persistent headache. Pharmacological treatments often consist of analgesics and non-steroidal anti-inflammatory drugs, tricyclic antidepressants, or antiepileptic medication. Cognitive behavioral therapy, relaxation techniques, biofeedback, and physical therapy could also be used for PTH treatment. Our work highlighted the need for more rigorous studies to better describe the importance of identifying risk factors and patient-centered treatments and to evaluate the effectiveness of the existing treatment options. Clinicians should consider a multidisciplinary approach to managing PTH, including pharmacotherapy, cognitive behavioral therapy, and lifestyle changes.

## 1. Introduction

Post-traumatic headache (PTH) is a common and debilitating consequence of mild traumatic brain injury (mTBI). Despite its high prevalence and significant impact on daily functioning and quality of life, PTH remains a complex and poorly understood phenomenon. Several controversies also address the prevalence rates of PTH [1,2,3]. Moreover, a significant proportion of individuals suffering from PTH continue to experience symptoms one year after the injury, indicating chronic PTH [4]. This highlights the need for a better understanding of the underlying pathophysiology and risk factors to facilitate early identification and management of PTH. 

The pathophysiological mechanisms underlying PTH remains not fully understood; however, both migraine and TBI mechanisms seem implicated in the impaired descending pain modulation, neurometabolic changes, neuroinflammation, cortical spreading depression, and release of the calcitonin-gene-related peptide (CGRP) seen in both pathologies [5]. The development and persistence of PTH may be more related to neuroinflammation and trigeminal system activation implicated in migraine and other primary headache disorders than to the mechanisms underlying TBI [5]. TBI can lead to immediate effects of brain concussion, cerebral blood vessel damage, and axonal shearing, as well as a secondary cascade of metabolic and cellular excitotoxic and inflammatory changes that can promote the development of PTH [6]. Despite this, the relationship between TBI mechanisms and PTH was not clearly established. The similarities between PTH and migraine are supported by the observation that patients with PTH who have no prior history of migraine exhibit hypersensitivity to CGRP [7]. CGRP is a neuropeptide that may mediate trigemino-vascular pain transmission and trigger a migraine attack [8,9]. CGRP antagonists are a class of medications used in the acute and preventive treatment of migraine. A study of patients with PTH found that 28% had a 50% reduction in days with moderate or severe headache following open-label treatment with erenumab, a CGRP receptor antagonist [10] (Table 1).

Despite extensive research, there is limited compelling evidence regarding risk factors for acute or persistent PTH [12]. However, several risk factors have been identified in small observational series of patients with PTH [13,14,15,16,17]. These risk factors include: age ≤ 60 years [13,16], female sex [14,15], mTBI (versus moderate or severe TBIs), history of prior TBI, history of prior headaches [15,18], and comorbid psychiatric conditions [14] (Table 2).

Additionally, several studies have investigated the role of early headache as a risk factor for PTH. Babcock et al. found that adolescents who addressed the Emergency Department with a headache after mTBI exhibited a high risk to develop PCS (post-concussion syndrome), including PTHs [18]. Root et al. reported that early PTH, or previous history of headache, was positively associated with an increased risk of prolonged PCS and persistent PTHs [19]. Headache was the only individual symptom that significantly predicted worse attention/executive functioning performance in the study by Guty et al. [20]. None of the other symptoms were significantly related to memory performance, but a longer period since injury was related to worse memory performance [20]. However, not all studies have found a significant association between early headache and the development of persistent PTH [1]. 

Womble et al. [26] found that the female sex was a risk factor for vestibular–oculomotor symptoms/impairment after sport-related concussion that also include PTHs. Moreover, Ahman et al. [21] found that female sex was a significant risk factor for PTH at three months post-injury, with 57% of females reporting headaches compared to 41% of males. Similarly, Ingebrigtsen et al. [28] reported that females were found to have a higher prevalence of PCS overall, including headaches. 

Lu et al. reported that healthcare providers identified several risk factors for PTHs, including visual changes, nausea/vomiting, neck pain, and pre-existing conditions, such as migraines [22]. Wilk et al. [24] found that soldiers who experienced loss of consciousness (LOC) due to a blast mechanism of concussion were more likely to report headaches and tinnitus 3 to 6 months post-deployment compared to those with a non-blast mechanism.

Wilk et al. [26] reported that soldiers who experienced multiple mTBIs with LOC were at an increased risk of developing headaches, compared with those who experienced a single occurrence. Ferdosi et al. [25], while analyzing data from a cohort of recently deployed soldiers, found that individuals with a recent history of mTBI were more likely to endorse one or more PCS as “severe” and/or “very severe”, compared with those without mTBI. The prevalence of clinically relevant PCS remained relatively constant over one year of follow-up, whether or not symptoms were associated with a concussion.

Wilk et al. reported that multiple deployment-related TBIs were collectively and/or individually associated with depression, anxiety, post-traumatic stress disorder (PTSD), and PCS [23]. The same authors in another study, after adjusting for PTSD, depression, and other factors, LOC was significantly associated with three post-concussive symptoms, including headaches [28]. However, these symptoms were more strongly associated with PTSD and depression than with the history of mTBI. PTSD is also a major risk factor of PTH development following a mTBI and could lead to severe headaches due to the psychological burden of the experienced physical trauma underlying the mTBI [29].

Vanderploeg et al. found that combat exposures with and without physical injury were each associated not only with PTSD, but were not specifically associated with post-concussive symptoms [11]. The experience of seeing others wounded or killed or experiencing the death of a buddy or leader was associated with indigestion and headaches, but not with depression, anxiety, or PTSD.

Further research is needed to fully elucidate the relationship between PTSD, depression, and PTHs, as the previous studies suggested that there may be a link between these conditions. It is important for clinicians to screen for and appropriately treat these comorbid conditions in individuals with PTHs to improve outcomes.

Early symptoms indicative of post-traumatic migraines, such as headache, nausea, light sensitivity, and noise sensitivity, were associated with experiencing vestibular–oculomotor symptoms/impairment after sports-related concussions [24]. Lu et al. [22] surveyed healthcare providers about their evaluation and treatment of mTBI in adults. The study found that providers focused on assessing visual changes, nausea/vomiting, headache, and neck pain, but did not mention assessing common post-concussive symptoms of fatigue, emotional changes, and sleep impairments [25]. These studies suggested that a history of headache at the time of injury, female sex, pre-existing conditions, blast mechanism of concussion, post-traumatic migraine symptoms, multiple mTBIs, and comorbid PTSD and depression are important risk factors to consider when evaluating patients for PTHs.

Kraemer et al. [27] assessed the prevalence of psychiatric disorders at three to four months after mTBI and found that the frequency of psychiatric disorders was not correlated with the occurrence of persistent PTH, suggesting that pre-existing psychiatric disorders may not be a significant risk factor for PTH. However, further research is needed with respect to this issue. Insomnia, vertigo, older age, and somatic pain have also been reported as risk factors for PTH [27]. 

## 2. Prevalence of Post-Traumatic Headaches

Following mTBI, PTH is the most commonly reported symptom [30]. A wide range of variation for the prevalence of PTH in the general population (30% to 90%) was reported after mTBI events due to the methodological differences, study population descriptions, and follow-up periods [1]. The incidence of PTH is primarily based on data obtained from patients who seek immediate medical attention at emergency clinics or trauma centers. The incidence of PTH in patients who seek medical help several days after trauma, typically through general practitioners, is, however, hard to estimate. Furthermore, PTH may be misdiagnosed as migraine or other primary headache syndromes, particularly in those with a history of headaches. The prevalence of PTHs varies across different studies, likely due to differences in study design, populations, and methods of assessing headaches. 

Globally, approximately 69 million individuals suffer from TBI every year, with mTBI being the most frequent severity-based subtype [31]. Observational studies indicated that PTH is reported in 37 to 69 percent of patients with mTBI [13,14,32,33]. Patients may exhibit short-term PTH symptoms that resolve within three months of injury [13,14], while others may experience chronic PTH symptoms. In one cohort, 32 and 29 percent of patients reported persistent PTH after three and six months, respectively, following mTBI [34]. Furthermore, other studies have demonstrated that 49 to 58 percent of patients continue to experience PTH 12 months after injury [13,35]. The lifetime prevalence of PTH was 4.7 percent in males and 2.4 percent in females in a Danish-population-based study [36,37], whereas in Norway, only 0.21 percent of patients with chronic headache had persistent PTH [36]. 

In pediatric and adolescent populations, the prevalence of PTH following a concussion is estimated to be between 43% and 60% [3]. However, a recent study [38] that evaluated the incidence, clinical characteristics, and risk factors of PTH in children (age < 14 years) found that PTH occurred in only 13% of the recruited children that suffered from mild head impacts caused by car accidents, falls, play or sports-related head bumps or head struck by objects. However, van Ierssel et al. [39] provided a much higher prevalence for PTH, as out of their 548 children with concussion, 71.8% suffered from PTH. Another study evaluating pediatric PTH reported a prevalence of 33% from the TBI cases of adolescents (age < 19 years) that suffered from sport injuries, vehicles accidents, and falls [40]. Thus, similar to adult patients, the prevalence of PTH in children could also widely vary.

Furthermore, the incidence of persistent PTH appears to be inversely related to the severity of TBI. Specifically, PTH is found in 58% of patients with mTBI 12 months after the initial injury compared to 33% of patients with moderate to severe TBI [41]. Similar trends were reported over the years by many studies [30]. In a recent animal study, Bree et al. [42] discussed this paradox yet not elucidated and suggested that a pain hypersensitivity phenotype could be developed as a result to more severe head trauma, as compared to other rat models of milder TBIs, arguing thus the clinical findings. In this context, this trend could be the result of some bias that the prospective studies failed to address during experimental design.

Athletes and military personnel with concussions are both at risk for PTH, although the incidence in these groups may be underreported [43]. In athletes, the prevalence of PTH has been reported to be as high as 93% after sports-related concussion and it is thought that this is the most common PCS symptom [1,2]. Most of the reports agree in regarding the prevalence of PTH in sports-related concussion [44,45,46]. Moreover, even amateur athletics activities were reported to produce TBIs (in almost 20% of the adults) [13]. On the other hand, Ingebrigtsen et al. [28] reported that 62% of the included patients that suffered from mTBI caused by traffic accidents, falls, sports injuries, or assaults but had normal brain computed tomography scan (CT) presented at least 1 post-concussion symptom, while 40% of the patients fulfilled the diagnostic criteria for PCS which is basically an mTBI of which symptoms last for several weeks or months after the injury that caused the mTBI. PTH was most often reported, along with irritability, anxiety, dizziness, fatigue, and impaired concentration, PCS being the most common risk factor of PTH.

Wilk et al. [23] examined the prevalence of persistent PCS in 587 US Army infantry soldiers who met the criteria for concussion following a deployment to Iraq. Of those who lost consciousness, blast mechanism was significantly associated with headaches and tinnitus 3 to 6 months post-deployment, compared with non-blast mechanisms [23]. However, among the larger group of soldiers reporting concussions without LOC, blast was not associated with adverse health outcomes. Another study of the same group [24] included 1502 US Army soldiers who were administered surveys 4 to 6 months after returning from deployment to Iraq or Afghanistan and reported that LOC was significantly associated with headaches. Still, these symptoms were more strongly associated with PTSD and depression than a history of mTBI. Multiple mTBIs with LOC increased the risk of headache, compared with the unique occurrence of head trauma, although depression remained a strong predictor [24].

Triplett et al. [8] conducted a study on a non-clinical population of 1067 persons. They reported that headache was the most commonly reported of an array of symptoms that are essentially post-concussion symptoms, but the prevalence of PTH was not reported. Vanderploeg et al. [11] surveyed 3098 members of the Florida National Guard (1443 deployed, 1655 not deployed). They found that deployment-related mTBI was associated with PCS, with symptoms including headaches, depression, anxiety, and PTSD [11]. Additionally, combat exposure with and without physical injury was associated with PTSD and numerous PCS and non-PCS symptoms. However, PTH remained a common symptom following mTBI or concussion, with increased prevalence in individuals who experienced LOC or blast exposure [24,47]. Nevertheless, the prevalence of PTHs may also be influenced by other factors, such as pre-existing conditions, comorbidities, and individual differences in pain perception and processing. It is important for healthcare providers to consider these factors when evaluating and treating individuals with PTHs.

## 3. Clinical Profiles of Chronic Post-Traumatic Headache

Approximately 15–20% of individuals who experience concussion continue to suffer from headaches one year after the injury, indicating chronic PTH [4].

The clinical features of PTH vary, but often resemble those of primary headache syndromes. Most commonly, following mTBIs are reported migraine-like and tension-type-like headache types [13,43,48,49,50], with the predominance of the latter (75 to 77%) [49,50]. Similar findings were reported for children, the ratio of headache types was 1:3 (tension-type headaches:migraines) [38]. However, in other studies of both adults and children, the predominance of migraine-type headaches was reported [13,39,48]. Furthermore, it was shown that in mTBI patients experiencing PTH associated with blast trauma, most headaches were migraine-like types [51]. In athletes with TBI, the most prevalent type of headache reported was migraine [52].

Nevertheless, many patients (27 to 75%) experienced more than one type of headache [52,53]. The frequency of headache burden may vary, with a mean frequency of 25 days per month reported in one study of 100 patients with PTH [49]. In rare cases, PTH symptoms may mimic other primary headache disorders, such as trigeminal autonomic cephalgias, such as cluster headache [53], short-lasting unilateral headache with cranial autonomic symptoms [54], and paroxysmal hemicrania [55], as well as hemicrania continua [56]. Distinguishing whether the headache pattern is migraine-like, tension-type-like, or resembling other headache disorders can guide symptomatic therapy [57]. In one study, acute PTH was frequently characterized by bilateral localization (56%), moderate or severe intensity (59%), and pressing quality (69%) [58].

Multiple triggers and aggravating factors of PTH have been reported. Patients with persistent PTH and migraine-like symptoms reported that bright lights exacerbate headaches, while those with tension-type-like headache symptoms reported stress and nervousness as headache-aggravating factors [33]. By comparison, another study found that patients with migraine-like headache symptoms reported stress (73%), lack of sleep (69%), and bright lights (60%) as headache-aggravating factors [48]. Theeler et al. found that 58% of PTH in military personnel were classified as migraine [58], while Ducic et al. reported that 25% of their PTH patients had tension-type headaches [59].

Non-specific PTH is another subtype linked explicitly to head injury being defined as a headache that develops within seven days of a head injury or concussion and persists for over three months [60]. While most of the headache attacks resemble migraines or tension-type headaches, other symptoms, such as dizziness, neck pain, and cognitive difficulties, were reported.

Chronic daily headache (CDH) is another subtype of headache that may develop after a head injury. CDH is defined as a headache that occurs for more than 15 days per month for at least three months. CDH can be further classified into subtypes, including chronic migraine and chronic tension-type headache [61].

Patients with PTH may also exhibit other symptoms of PCS, including fatigue, dizziness, sleep disorders, concentration difficulties, and seizures. Depression, anxiety, and insomnia frequently occur after mTBI and PTH [62,63]. Additionally, PTSD could be present in more than 10% of patients after TBI [64].

One important characteristic is the timing of the onset of the headache which can be either immediate or delayed. Immediate PTH typically starts within seconds or minutes of the traumatic event and can last up to 7 days. Delayed PTH, on the other hand, can develop hours, days, or even weeks after the initial injury and can persist for several months or even years.

Another essential clinical profile is the intensity and frequency of the headache. PTHs are commonly described as mild to moderate pressure or tension-type headache, but can also be severe and debilitating. The frequency of headaches can vary from occasional to daily and can be persistent or intermittent (Table 3).

In some cases, PTH can be a part of a more complex PCS, which can include a range of symptoms such as fatigue, sleep disturbances, and mood changes. It is important to note that the classification and diagnosis of headache subtypes in PTH can be challenging, as there is often significant overlap in symptom presentation between different headache subtypes. As such, a comprehensive evaluation and individualized treatment plan tailored to the patient’s specific headache symptoms and characteristics are crucial for effectively managing PTH.

## 4. Diagnosis of Post-Traumatic Headache

The diagnosis of PTH is based on symptomatic evaluation of a patient who meets the diagnostic criteria for TBI. PTH is classified according to the International Classification of Headache Disorders (ICHD-3) as either acute or persistent and attributed to either mTBI or moderate to severe TBI [65].

The key criteria for diagnosis are a history of head trauma, the onset of headache within seven days of trauma, and a headache syndrome not better accounted for by another headache diagnosis. Despite the fact that the seven-day interval might be subjective in some cases, it may be useful in excluding patients with isolated acute headaches who do not subsequently develop PTH.

Acute PTH may last up to three months; for prolonged symptoms, PTH could be considered chronic or persistent [65]. Imaging or laboratory test findings are not useful in identifying patients with or without PTH following TBI. However, imaging studies may be necessary to rule out intracranial hemorrhages, such as parenchymal bleeding, epidural or subdural hemorrhages, and skull fractures [65,66]. Several brain imaging and PTH-associated exploratory assessments revealed that PTH is associated with abnormalities in brain structure and functions, including reduced cortical thickness in the frontal and parietal regions [67,68]. Additionally, patients with persistent PTH show differences in brain volume, surface area, fiber tract integrity, and functional connectivity, compared with patients diagnosed with migraine [69,70,71]. Additionally, some molecular biomarkers, such as S100 calcium-binding protein B and calcitonin-gene-related peptide (CGRP), were found altered in patients with persistent PTH [72,73].

Additional criteria may be applied to subcategorize patients with PTH according to the severity of TBI. For PTH attributed to moderate or severe TBI, the patients exhibit LOC for >30 min, post-traumatic amnesia or altered level of awareness for more than 24 h, receive a Glasgow coma scale (GCS) score < 13, or brain imaging assessment is suggestive for TBI (skull fracture, intracranial hemorrhage, or brain contusion) [65]. On the other hand, PTH is attributed to mTBI when the headache is also associated with transient confusion, disorientation, or impaired consciousness; short term memory loss (events immediately before or after the head injury), or two or more symptoms of mTBI, such as nausea, vomiting, visual disturbances, dizziness or vertigo, gait imbalance, or impaired cognitive functions.

The alternative causes of headache should be further evaluated based on patient history and clinical examination for atypical symptoms or neurologic deficits that could suggest alternative diagnoses and additional testing. As standard examination of acute TBI patients often includes head CT or MRI during the initial evaluation, the patients with PTH may have already undergone neuroimaging evaluation. In patients with aggravated symptoms, brain MRI with gadolinium contrast is preferred. Other diagnostic testing, such as electroencephalogram (EEG) or lumbar puncture, is reserved for patients with atypical symptoms or abnormal examination findings suggestive of alternate diagnoses. Several other headache syndromes may occur after TBI, including intracranial hypotension, subdural hematoma, occipital neuralgia, trigeminal neuropathy, cervical artery dissection, temporomandibular joint disorders, premorbid headache syndromes, and medication overuse headache. Differentiation from PTH is based on specific headache symptoms and associated neurologic findings [74,75,76,77].

## 5. Treatment

### 5.1. Pharmacological Treatments

There are several pharmacological treatments available for PTHs. Nonsteroidal anti-inflammatory drugs (NSAIDs), such as ibuprofen and naproxen, are commonly used to manage mild to moderate headaches. Paracetamol can also be effective in managing headaches.

In this way, the association between acute administration of ibuprofen, acetaminophen, or both and the resolution of headache or reduction in headache pain at seven days post-concussion in children and youth was previously investigated [75]. It was found that 74.3% of the individuals aged 5–18 years with acute concussion that were recruited manifested acute headache upon ED presentation; of these, 67.8% received analgesic medication before or during their ED visit. Multivariate analysis pertained to 1707 participants with propensity scores based on personal characteristics and symptoms, and 51.4% reported headache at seven days post-concussion, which was the primary outcome. While using propensity scores and adjusted multivariate regression models, no association between acute administration of ibuprofen, acetaminophen, or both, and headache presence at seven days was obtained, suggesting that the acute treatment with ibuprofen, acetaminophen, or both in the acute phase of TBI could not decrease the risk of headache seven days post-concussion. Despite this, non-opioid analgesics, such as ibuprofen or acetaminophen, could be prescribed for short-term headache relief. However, caution is advised in analgesics long term prescription and use when headache symptoms persist [3].

Other useful medication in PTH could be antiepileptic drugs and tricyclic antidepressants (TCAs). Cushman et al. [76] conducted a retrospective study to examine the correlation between medication (gabapentin and TCAs) and concussion-associated symptoms reduction. While the patients diagnosed with clinical concussion self-reported their symptom scores based on the Post-Concussion Symptom Scale (PCSS), some received prescription for gabapentin or TCAs for headache treatment and were followed-up for over one year post-concussion. The mixed-effects analysis showed a significant decrease in headache and symptom scores over time in each medication group and in those not receiving medication (*p*  ≤  0.014 for all scenarios, B = −0.005 and −0.08, respectively). Although patients in the medication groups showed significantly higher headache and symptom scores (*p*  <  0.001), the results showed that medication significantly affected longitudinal improvements in the outcome scores. However, the piecewise regression analysis showed short-term improvements with gabapentin (1.3 points, *p*  =  0.004) and more sustained improvements with TCAs (3.5 points, *p*  =  0.006), suggesting the immediate effects of gabapentin and TCAs on improving symptom burden, despite the fact that the long-term outcomes show similar improvement compared with those who are not prescribed medication.

Acute and prophylactic medical therapies efficiency for chronic PTH attributable to mTBI was also evaluated. In a retrospective cohort study that recruited US Army soldiers that were exposed to occupational mild head trauma that were followed for three months after starting headache prophylactic medication, Erickson et al. [77] compared the treatment outcomes by relation to blast and non-blast-related PTH and found that the headache frequency among all PTH subjects decreased from 17.1 days/month at baseline to 14.5 days/month at follow-up (*p* = 0.009). Additionally, a significant decline in headache frequency occurred in subjects treated with topiramate (*n* = 29, −23%, *p* = 0.02), but not among those treated with a low-dose TCAs (*n* = 48, −12%, *p* = 0.23). Seventy percent of PTH patients who used triptan class medication experienced reliable headache relief within 2 h compared to 42% of subjects using other headache abortive medications (*p* = 0.01). Moreover, the results showed that triptan-based medications were effective for both blast PTH and non-blast PTH (66% response rate vs. 86% response rate, respectively; *p* = 0.20). It was also shown that headache frequency could decrease in 41% of non-blast PTH compared to 9% among blast PTH, and that 57% of non-blast PTH subjects had a 50% or greater decline in headache frequency compared to 29% of blast PTH subjects (*p* = 0.023). Headache-related disability, as measured by mean Migraine Disability Assessment Scores, declined by 57% among all PTH subjects, with no significant difference between blast PTH (−56%) and non-blast PTH (−61%) suggesting that chronic PTH triggered by a blast injury may be less responsive to commonly prescribed headache prophylactic medications than non-blast PTH. In this context, it was concluded that triptan-class medications are effective for treating headaches due to blast or non-blast-related chronic PTH and that topiramate could be effective in headache prophylaxis in chronic PTH patients.

While no definitive pharmacological treatments exist for persistent PTH, peripheral nerve surgery may be a safe and effective option for some patients. It is crucial to consider an individualized treatment approach, considering the patient’s specific symptoms and medical history and the potential risks and benefits of various treatment options. A multidisciplinary approach involving healthcare providers from different specialties is also beneficial for evaluating and managing persistent PTH.

### 5.2. Repetitive Transcranial Magnetic Stimulation

Repetitive transcranial magnetic stimulation (rTMS) is a non-invasive neurostimulation technique that has been investigated as a potential treatment for PTH. In a systematic review of 11 prospective rTMS treatment studies of patients with mild TBI/concussion, Mollica et al. found promising preliminary results for rTMS in treating post-concussion depression and PTH, two symptoms that otherwise have limited effective treatment options [78]. Positive results were found in two out of four studies with depression as a primary outcome and in all three studies that assessed depression as a secondary outcome. All four rTMS studies for PTH reported positive results. Despite the fact that all the studies were based on small pilot samples and the methodological heterogeneity precluded a quantitative meta-analysis, the results suggested that rTMS may be a promising treatment modality for PTH.

### 5.3. Neutralizing Prismatic Lenses

Neutralizing prismatic lenses have been investigated as a treatment modality for PTH in patients with vertical heterophoria (VH). Rosner et al. conducted a retrospective study of 38 patients with persistent post-concussive symptoms and VH diagnosed by an optometric binocular vision sub-specialist [79]. The patients were fitted with neutralizing prismatic lenses, and data were collected before and after prism, an application using validated survey instruments for headache, dizziness, anxiety, and BVD symptom burden, as well as subjective ratings of headache, dizziness, and anxiety severity. A sub-analysis of the BVD survey instrument questions that pertain specifically to headache, dizziness, and anxiety was also conducted. The results demonstrated a marked reduction in all measures of headache, dizziness, and anxiety (19.1–60.8%) and an overall subjective improvement of VH symptoms of 80.2%, suggesting that neutralizing prismatic lenses may be an effective treatment for PTH in patients with VH.

### 5.4. Peripheral Nerve Surgery

Peripheral nerve surgery has been investigated as a safe and effective treatment possibility for PTH in patients with chronic pain that persists despite initial treatments by a neurologist specialized in headache management. Ducic et al. conducted a retrospective review of 28 consecutive patients with PTH who underwent occipital nerve surgery [59]. Preoperative and postoperative headache pain was evaluated on a visual analogue scale (VAS) in 24 patients with at least six months of follow-up. The average VAS headache pain reduced from 6.4 preoperatively to 1.4 (*p*  <  0.0001), and 21 patients (88%) had a successful outcome of at least a 50% reduction in their VAS following peripheral nerve surgery. Additionally, 12 patients (50%) were pain-free during the final follow-up and no surgical complications occurred.

### 5.5. Botulinum Toxin

Maria Lippert-Grüner reported the case of a 62-year-old woman with a history of major TBI who developed chronic tension-type PTHs that were not relieved by oral medication [80]. The patient received local injections of 22 IU Botox^®^ into the frontalis and corrugator supercilii muscles. Headaches improved after five days and were completely resolved after ten days, even during stressful situations, suggesting that Botulinum toxin injection might be an effective treatment for PTH.

Yerry et al. conducted a retrospective study of onabotulinum toxin A to treat PCS [81] and showed that the patients receiving the toxin manifested significant reduction in headache frequency, intensity, and medication usage. The study also found improvements in cognitive function and quality-of-life measures. However, the study did not include a control group and was limited by its retrospective design.

## 6. Prognosis

Due to its heterogeneity, the prognosis of PTH is poorly understood [82,83]. Some patients with acute PTH do not develop persistent PTH, and headaches may resolve spontaneously or with treatment within three months of the TBI [82]. However, for others, PTH may be chronic, while the natural history of PTH is not well described [84]. Recent data suggested that the recovery from PTH is slower when headaches have migraine-like features [82]. In one study of 110 patients with mTBI, 76% reported headaches after one month, and 58% reported persisting PTH at a mean follow-up of 98 days [84]. Another study found that only 30% of 116 patients with PTH reported headache resolution by 24 months after TBI [83]. These findings could suggest that the persistence of PTH for some patients after TBI may reflect secondary chronic post-inflammatory and metabolic changes related to migraine pathophysiology [24].

It is worth mentioning that PTH patients could also exhibit other PCS symptoms which typically resolve within 3 to 12 months in most cases [83]. However, the prognosis for PTH is specifically variable and may be chronic for some patients [82,84,85]. Further research is needed to better understand the natural history and long-term outcomes of PTH.

## 7. Conclusions

Overall, the present study highlighted the need for further studies to better describe the importance of identifying risk factors and patient-centered treatments, along with the need to evaluate the effectiveness of the existing treatment options. Clinicians should consider a multidisciplinary approach to managing PTH, including pharmacotherapy, cognitive behavioral therapy, and lifestyle change.

## Figures and Tables

**Table 1:** Summary of pathophysiological mechanisms involved in PTH pathogenesis.

Pathophysiology Features	Description
Impaired descending pain modulation [5]	Abnormalities in the central nervous system’s pain control system leading to an increase in pain sensitivity.
Neurometabolic changes [5]	Changes in the brain’s metabolic activity, including an increase in lactate and a decrease in glucose metabolism, leading to a cascade of neuroinflammatory events.
Neuroinflammation [5]	A response to the injury that leads to an increase in pro-inflammatory cytokines and chemokines and activation of microglia and astrocytes.
Cortical spreading depression [5]	A wave of depolarization that spreads across the cortex leading to the release of inflammatory molecules and a decrease in cerebral blood flow.
Release of calcitonin gene-related peptide (CGRP) [5]	A neuropeptide that may mediate trigeminovascular pain transmission and trigger a migraine attack.
Trigeminal system activation [5]	Activation of the trigeminal nerve, which is implicated in migraine and other primary headache disorders.
Secondary cascade of metabolic and cellular excitotoxic and inflammatory changes [6]	TBI can lead to a secondary cascade of events that can promote the development of PTH.
Hypersensitivity to CGRP [9,11]	Patients with PTH who have no prior history of migraine exhibit hypersensitivity to CGRP.
Erenumab treatment [10]	A CGRP receptor antagonist that has been shown to reduce the number of days with moderate or severe headache in patients with PTH.

**Table 2:** Summary of main risk factors for PTH.

Risk Factor	Description
Headache at injury	Early post-injury headache or a previous history of headache are positively associated with prolonged PCS and persistent PTHs [18,19,20].
Female sex	Female sex is a risk factor for PTHs [21,22].
Pre-existing conditions	Pre-existing conditions, such as migraines, are risk factors for PTHs [22].
Blast mechanism	Loss of consciousness due to a blast mechanism of concussion is associated with an increased risk of headaches [23].
Multiple mTBIs	Multiple mTBIs with loss of consciousness are associated with an increased risk of headaches [24,25].
PTSD and depression	There is a link between PTSD, depression, and PTHs [8,11,23,24].
Post-traumatic migraine symptoms	Early symptoms indicative of post-traumatic migraines are associated with PTHs [26].
Insomnia	Insomnia was reported as a risk factor for PTHs [27].
Vertigo	Vertigo was reported as a risk factor for PTHs [27].
Older age	Older age was reported as a risk factor for PTHs [27].
Somatic pain	Somatic pain was reported as a risk factor for PTHs [27].

**Table 3:** Summary of clinical features of PTH.

Clinical Features of Post-Traumatic Headache (PTH)
PTH most commonly resembles migraine-like and tension-type-like headaches [13,19,20,21,39];
Tension-type-like headaches are the most frequent in most PTH patients (75 to 77%) [40,41]; however, in others, migraine-type headaches predominate [13,19,22];
In individuals with PTH associated with blast trauma most headaches were migraine-like [22];
Many patients (27 to 75%) experience more than one type of headache [25,26];
Rarely, PTH symptoms mimic other primary headache disorders, such as trigeminal autonomic cephalgias, cluster headache [27], short-lasting unilateral headache with cranial autonomic symptoms [48], and paroxysmal hemicrania [49], as well as hemicrania continua [50];
Multiple triggers and aggravating factors of PTH have been reported: ○ bright lights (in patients with persistent PTH and migraine-like headache symptoms) ○ stress and nervousness (in patients with tension-like headache symptoms) [50];
Acute PTH was frequently characterized by a bilateral localization (56%), moderate or severe intensity (59%), and pressing quality (69%) [51];
Other symptoms of PCS could co-occur with PTH: fatigue, dizziness, sleep disturbances, concentration difficulties, and seizures;
Depression, anxiety, and insomnia frequently occur after TBI and PTH [55,56];
PTH may be classified into non-specific PTH, chronic daily headache (CDH), chronic migraine, and chronic tension-type headache [53,54].

## Data Availability

All data is available on request.
